# Nomogram for predicting spontaneous pregnancy after microscopic varicocelectomy in infertile men with normal hormone

**DOI:** 10.1186/s12884-022-05125-9

**Published:** 2022-10-26

**Authors:** Lina Liu, Jia Li, Gang Liu, Chunyu Pan, Song Bai, Yunhong Zhan, Liping Shan

**Affiliations:** 1 Department of General Surgery, Shengjing Hospital of China Medical University, Shenyang, China; 2 Department of Urology, Shengjing Hospital of China Medical University, No. 36, San Hao Street, Shenyang, Liaoning, 110004 China

**Keywords:** Varicocele, Varicocelectomy, Spontaneous pregnancy, Nomogram, Predictor

## Abstract

**Introduction:**

The current challenge for the treatment of varicocele is identifying patients who could benefit the most from surgery. We aimed to develop and validate a nomogram for predicting spontaneous pregnancy following microscopic varicocelectomy in infertile men, based on a large cohort.

**Methods:**

Two hundred eighty-two consecutive patients who underwent microscopic varicocelectomy from January 2018 to December 2020 were enrolled as participants in the study. Xiang Hua center (206 patients) as a development cohort. Hu Nan center (76 patients) as a validation cohort. Patient clinicopathologic data were recorded. Multivariate logistic regression was used to build a predictive model with regression coefficients. Then, backward stepwise selection was applied, and the likelihood ratio test with Akaike’s information criterion was used as the stopping rule. The performance of this predictive model was assessed for discrimination, calibration, and clinical usefulness.

**Results:**

Predictors of this model included the age of female partners, diameter of veins, initial and increased total progressively motile sperm count. The model demonstrated good discrimination with an AUROC of 0.925 (*p* < 0.001) and calibration (Unreliability test, *p* = 0.522) in the validation cohort. Furthermore, the model was clinically useful, according to decision curve analysis.

**Conclusions:**

Our findings indicated that younger female partners, larger diameter of veins, higher initial and increased total progressively motile sperm count were significant predictors of spontaneous pregnancy in infertile men, post microscopic varicocelectomy. This nomogram may assist in individual decision-making on the treatment strategy of varicocele preoperatively and improve the treatment outcome.

## Introduction

Varicocele is characterized by abnormal dilatation of testicular veins in the pampiniformis plexus form and is caused by impaired venous drainage [1]. It can progressively affect male fertility, testicular growth, and lead to symptoms of scrotal pain and discomfort, even hypogonadism [2]. Varicocele is present in 15% of men with normal semen quality, 25% with abnormal semen quality, and 35–40% of infertile men [3].

Varicocelectomy is the most frequently performed procedure. Recent RCTs and meta-analyses revealed that varicocelectomy could improve semen parameters, spontaneous pregnancy (SP) rates, and the results of assisted reproductive technologies [4–6]. Several techniques were used, including microscopic, laparoscopic and open surgery, and interventional embolization [7]. The ideal varicocele treatment strategy should combine excellent efficacy and safety with minimally invasive techniques. Therefore, microscopic varicocelectomy (MSV) has gradually become the preferred surgical option due to its advantages which include meticulous ligation of the veins while sparing the arterial branches, lower postoperative recurrence rate, fewer complications, higher postoperative semen quality and postoperative fertility rate [8–10]. MSV can be performed using either the inguinal or the subinguinal approach; both are effective methods for repairing varicocele in infertile men [11]. Subsequent to improving semen parameters, SP is considered the best indicator of a successful varicocelectomy. However, varicocelectomy improves semen parameters in 60–70% of patients only and fertility in 40–60% [12, 13]. The current challenge is determining which patients could benefit most from surgery.

There are only two retrospective studies addressing this issue. Shomarufov et al. conducted a retrospective study on 93 infertile men with varicocele and pathozoospermia who underwent MSV. They identified age, initial total sperm motility, and postoperative increase in total progressively motile sperm count (TPMSC) as predictors of SP post-surgery [14]. Another retrospective study included 176 infertile patients treated with subinguinal MSV. The results demonstrated that the incidence of improvement for sperm concentration and forward motility was 75.2%, only 45.5% of patients achieved SP at a mean follow-up of 11.7 months; A higher initial sperm concentration was a predictor of SP [15]. However, there is scarce and conflicting data on the prediction of SP post varicocelectomy. Therefore, no reliable recommendations can be made. In addition, none of these studies were validated by other cohorts. A nomogram derived from the predictive model is a reliable tool for predicting risk by incorporating and illustrating important predictors of significant clinical outcomes and yielding a numerical probability of the event. Therefore, this study aimed to develop and validate a nomogram for predicting SP post MSV in infertile men with abnormal semen parameters, based on a large cohort.

## Methods

### Study design

**Fig. 1:**
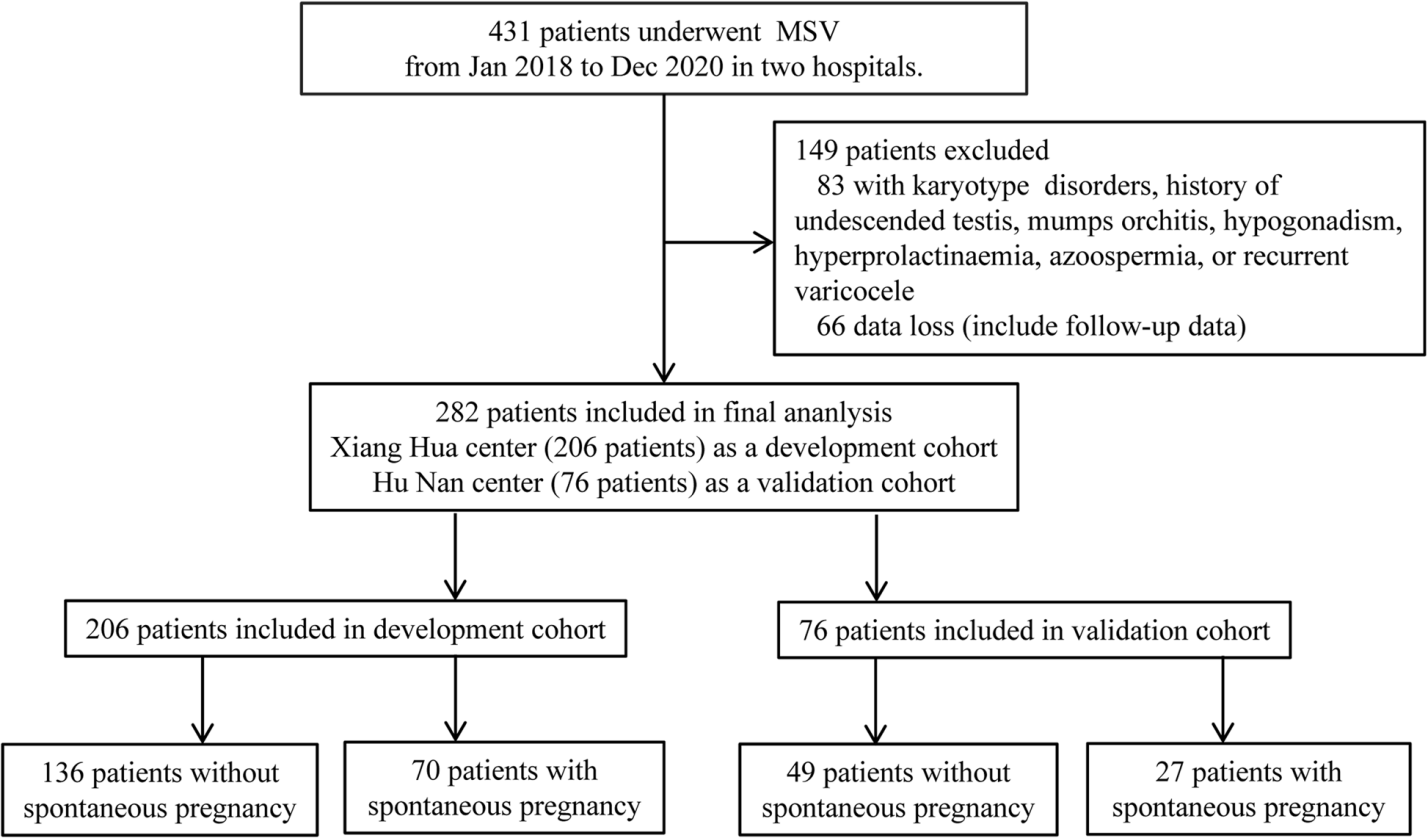
Flowchart of this study. Abbreviations: MSV, microscopic varicocelectomy

This retrospective study was conducted at Shengjing Hospital of China Medical University. It is the third-largest tertiary hospital in China with a high volume of patients with varicocele. A total of 431 consecutive patients who underwent microscopic varicocelectomy (MSV) from January 2018 to December 2020 were enrolled in the present study. After screening, there were 282 patients enrolled in the final analysis. Xiang Hua center (206 patients) as a development cohort; Hu Nan center (76 patients) as a validation cohort; see details in Fig. 1.

Ethical approval (2022PS719K) was provided by the Ethics Committee Shengjing Hospital Affiliated China Medical University. The clinical research registry UIN is ChiCTR2200060504 (03/06/2022). The study protocol conformed to the ethical guidelines of the 1975 Declaration of Helsinki.

### Inclusion and exclusion criteria

Inclusion criteria: patients who aged more than 18 years and underwent MSV (subinguinal or inguinal approach) due to clinically palpable varicocele; infertility with at least one abnormal semen parameter (concentration, total number, motility, or morphology); Patients who received infertility treatment (such as antioxidants, oestrogen receptor modulators and so on) during the peri-operative period.

Exclusion criteria: patients who had previous varicocelectomy (recurrent) or other types of inguinal surgery (such as hernia repair); chromosomal abnormality (karyotype disorders, or AZF microdeletions); malformation of the reproductive system (cryptorchidism); the presence of lower urinary tract infection or prostatitis, epididymitis, and seminal vesiculitis; the presence of hypopituitarism, hyperthyroidism, Cushing’s syndrome, or pituitary tumor; the presence of by ejaculation failure, retrograde ejaculation. Abnormal serum testosterone, follicle-stimulating hormone, luteinizing hormone; associated with female-factor infertility or unstable marriage.

The technique of microscopic varicocelectomy (MSV): Testicular artery and lymph vessels sparing and free ligation of gubernacular veins strategy were conducted.

### Measurement of characteristics and follow-up

Patient demographics (age, age of female partners, and body mass index [BMI]), infertility duration (months), smoke habitus (never vs. current or former), varicocele data (surgical side [left vs. Bilateral], grade [left, II vs. III], diameter of veins [left, mm], testicualr volume [left, ml]), semen parameters before MSV (baseline total progressively motile sperm count [TPMSC, 10^6^], sperm concentration, and morphology), intraoperative data (surgical approach [subinguinal vs. inguinal], number of ligated veins), semen parameters after MSV (increased TPMSC [10^6^], sperm concentration, and morphology), and decreased DNA fragmentation index (DFI).

The varicocele (VC) diagnosis was determined by the physical examination in the upright position. The VC was categorized based on the Dubin and Amelar grading system [16]**:** Grade 1: palpable during Valsalva maneuver. Grade 2: palpable at rest. Grade 3: visible and palpable at rest. All varicoceles were confirmed and measured by Doppler ultrasonography to detect the reflux of blood and their actual sizes (The maximum venous diameter). Infertility is defined as the absence of a desired pregnancy following regular, unprotected sexual intercourse for at least one year. A patient who had smoked 100 cigarettes in his lifetime is defined as currently smoking cigarettes. Testicular volume was measured by Lambert’s formula (V = L x W x H × 0.71). TPMSC (total progressively motile sperm count) was calculated from total sperm count × ratio progressively motile. Semen parameters after MSV were evaluated between three and six months after surgery. The difference between postoperative and preoperative TPMSC was used to define increased TPMSC. The difference between preoperative and postoperative DNA fragmentation index (DFI) (between three and six months after surgery) was used to define decreased DFI. Any SP that occurred was documented between six and twelve months after surgery. Oligozoospermia was defined as the decrease of sperm concentration less than 15 million sperm/mL. Semen analysis was performed and evaluated according to the World Health Organization criteria [17]. The follow-up assessment was conducted through clinic visits or telephone calls.

### Statistical analysis

IBM SPSS Statistics for Windows, version 22.0 (IBM Corporation), STATA 15.0 (Stata Corporation, College Station, TX, USA), and R software (version 3.0.1; https://www.r-project.org/) were used to analyze the data. In this study, the R packages 'rms' and 'glmnet' were employed. The statistical significance levels presented were all two-sided, with statistical significance defined as a probability (*P*) value of less than 0.05. To determine the normality of continuous variables, the Kolmogorov–Smirnov test was applied. The median (interquartile range) was used to represent non-normally distributed continuous variables, while the mean and standard deviation were used to represent normally distributed continuous variables. To compare the means of two continuous normally distributed variables, the independent-samples Student's t-test was utilized. The Mann–Whitney U test was used to compare two continuous non-normally distributed variables. Categorical variable was provided as number (percentage). The chi-squared and Fisher's exact tests were employed to compare categorical variables.

Multivariate unconditional logistic regression analysis was used for building a predictive nomogram with regression coefficients. The backward stepwise selection was applied using the likelihood ratio test with Akaike’s information criterion as the stopping rule [18, 19]. The model's performance was evaluated in a separate validation cohort. The validation cohort was given the logistic regression formula established in the development cohort, which was used to calculate the likelihood for each patient. To evaluate the model's discrimination performance, the area under the receiver operating characteristic (AUROC) curve was assessed. A 0.5 AUROC indicated no discrimination, whereas a 1.0 AUROC indicated complete discrimination. The model's calibration was evaluated using calibration plots, as well as the unreliability test and the Hosmer–Lemeshow (H–L) chi-square statistic (*P* > 0.05 indicates good calibration). Perfect calibration was indicated by a slope on the 45-degree line. The model's clinical usefulness was determined using decision curve analysis, which assessed the net benefits at various thresholds.

## Results

After screening with the same inclusion and exclusion criteria, 206 patients were included in the development cohort and 76 in the validation cohort. Of these, 34.0% (70/206) patients in the development cohort and 35.5% (27/76) in the validation cohort had SP, respectively; see details in Table 1.

**Table 1:** Demographics and clinical data in this cohort according to the development cohort and validation cohort

**Variables**	**Development cohort**	**Validation cohort**	***P* value**
Number of patients (%)	206	76	
**Demographic characteristics**			
Age (years)	31.27 ± 4.07	31.78 ± 3.90	0.351
Age, female partners (years)	29.68 ± 3.82	30.03 ± 3.53	0.491
BMI (kg/m^2^)	25.07 ± 3.82	25.38 ± 4.09	0.555
Infertility duration (months)	24.00 (12.00, 36.00)	24.00 (12.00, 36.00)	0.386
**Dietary habits**			
Smoke (never vs. current or former)	166 (80.60)/40 (19.40)	62 (81.60)/14 (18.40)	0.850
**Varicocele**			
Surgical side (left vs. bilateral)	118 (57.30)/88 (42.70)	41 (53.90)/35 (46.10)	0.616
Grade of palpation (left, II vs. III)	74 (35.90)/132 (64.10)	25 (32.90)/51 (67.10)	0.636
Diameter of veins (left, mm)	3.35 ± 0.30	3.35 ± 0.28	0.985
Testicular volume (left, ml)	15.80 ± 3.32	15.93 ± 3.17	0.754
**Semen parameters before MSV**			
Baseline TPMSC (10^6^)	10.74 ± 2.88	11.11 ± 2.80	0.328
Sperm concentration (10^6^/ml)	13.46 ± 4.24	13.59 ± 4.10	0.817
Oligospermia (yes)	116 (56.3)	40 (52.6)	0.581
Morphology (abnormal forms, %)	1.48 ± 0.50	1.41 ± 0.25	0.279
**Semen parameters after MSV**			
Increased TPMSC (10^6^)	16.83 ± 4.42	17.09 ± 4.57	0.668
Sperm concentration (10^6^/ml)	25.29 ± 4.26	24.99 ± 4.31	0.596
Morphology (normal forms, %)	4.92 ± 1.20	5.01 ± 1.28	0.581
Decreased DFI (%)	7.31 ± 2.15	7.20 ± 2.21	0.826
**Intraoperative data**			
Surgical approach (subinguinal vs. inguinal)	125 (60.70)/81 (39.30)	52 (68.40)/24 (31.60)	0.497
Ligated veins (number)	13.57 ± 4.59	13.70 ± 4.32	0.837

Continuous variables were expressed as median (interquartile range) or mean ± standard deviation

TPMSC = total sperm number (10^6^/ejaculate) × progressive motility (PR, %); PR, progressive (a + b motility); DFI, DNA fragmentation index

*Abbreviations: BMI* Body mass index, *MSV* Microscopic varicocelectomy, *TPMSC* Total progressively motile sperm count

**Fig. 2:**
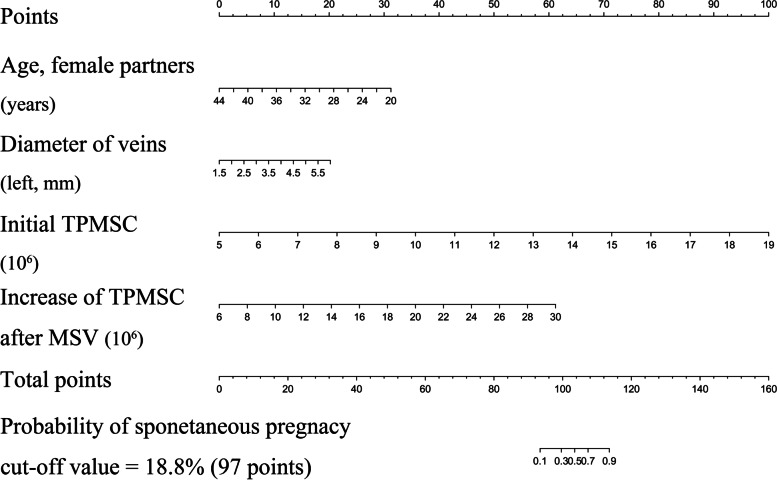
Nomogram to predict spontaneous pregnancy after MSV. Each clinicopathologic factor corresponds to a specific point by drawing a line straight upward to the Points axis. After summing the points located on the Total points axis, the sum represents the probability of experiencing spontaneous pregnancy after surgery by drawing a line straight down to the probability axis

In the univariate analysis of the development cohort, patients who experienced SP had more younger female partners, a larger diameter of veins, higher baseline TPMSC, and an increased TPMSC (Table 2). Multivariate binary logistic regression was used to build a predictive nomogram with regression coefficients. The backward stepwise selection was applied, and the likelihood ratio test with Akaike’s information criterion was used as the stopping rule. The results are presented in the final model (age of female partners, diameter of veins, baseline TPMSC, and increased TPMSC). Based on these results, we developed a predictive model, from which a nomogram predicting SP after MSV was generated (Table 3 and Fig. 2).

**Table 2:** Demographics and clinical data in this cohort according to the status of spontaneous pregnancy after MSV

**Variables**	**Development cohort** 206		***P* value**	**Validation cohort** 76		***P* value**
	**No SP**	**SP**		**No SP**	**SP**	
Number of patients (%)	136 (66.00)	70 (34.00)		49 (64.50)	27 (35.50)	
**Demographic characteristics**						
Age (years)	31.26 ± 4.26	31.29 ± 3.69	0.972	31.55 ± 3.91	32.19 ± 3.93	0.502
Age, female partners (years)	30.34 ± 3.89	28.40 ± 3.37	< 0.001	30.51 ± 3.44	29.15 ± 3.58	0.108
BMI (kg/m^2^)	25.16 ± 3.88	24.88 ± 3.72	0.621	25.61 ± 4.15	24.95 ± 4.02	0.503
Infertility duration (months)	24.00 (12.00, 36.00)	24.00 (12.00, 36.00)	0.990	24.00 (12.00, 36.00)	24.00 (12.00, 48.00)	0.204
**Dietary habits**						
Smoke (never vs. current or former)	114 (83.80)/22 (16.20)	52 (74.30)/18 (25.70)	0.101	43 (87.80)/6 (12.20)	19 (70.40)/8 (29.60)	0.072
**Varicocele**						
Surgical side (left vs. bilateral)	74 (54.40)/62 (45.60)	44 (62.90)/26 (37.10)	0.246	24 (49.00)/25 (51.00)	17 (63.00)/10 (37.00)	0.242
Grade of palpation (left, II vs. III)	46 (33.80)/90 (66.20)	28 (40.00)/42 (60.00)	0.381	12 (24.50)/37 (75.50)	13 (48.10)/14 (51.90)	0.036
Diameter of veins (left, mm)	3.22 ± 0.18	3.60 ± 0.45	< 0.001	3.27 ± 0.23	3.49 ± 0.34	0.075
Testicular volume (left, ml)	15.88 ± 3.56	15.63 ± 2.83	0.605	15.73 ± 3.35	16.30 ± 2.84	0.463
**Semen parameters before MSV**						
Baseline TPMSC (10^6^)	9.32 ± 2.01	13.48 ± 2.28	< 0.001	9.66 ± 1.98	13.74 ± 2.56	< 0.001
Sperm concentration (10^6^/ml)	13.46 ± 4.31	13.47 ± 4.14	0.980	13.22 ± 4.00	14.26 ± 4.26	0.295
Oligospermia (yes)	73 (53.7)	43 (61.4)	0.288	27 (55.1)	13 (48.1)	0.561
Morphology (abnormal forms, %)	1.49 ± 0.50	1.47 ± 0.50	0.851	1.37 ± 0.49	1.48 ± 0.51	0.339
**Semen parameters after MSV**						
Increased TPMSC (10^6^)	16.30 ± 4.26	17.85 ± 4.59	0.017	16.30 ± 4.70	18.51 ± 4.00	0.043
Sperm concentration (10^6^/ml)	25.31 ± 4.99	25.24 ± 4.79	0.913	24.63 ± 3.99	25.63 ± 4.86	0.338
Morphology (normal forms, %)	4.97 ± 1.19	4.83 ± 1.24	0.424	5.00 ± 1.29	5.04 ± 1.29	0.905
Decreased DFI (%)	7.10 ± 2.09	7.71 ± 2.24	0.057	7.21 ± 2.21	7.66 ± 2.25	0.399
**Intraoperative data**						
Surgical approach (subinguinal vs. inguinal)	84 (61.80)/52 (38.20)	48 (68.60)/22 (31.40)	0.335	35 (71.40)/14 (28.60)	17 (63.00)/10 (37.00)	0.447
Ligated veins (number)	13.81 ± 4.60	13.10 ± 4.56	0.290	14.22 ± 4.28	12.74 ± 4.30	0.153

Continuous variables were expressed as median (interquartile range) or mean ± standard deviation; categorical variables were reported as number (percentage). Independent samples Student's *t*-test was used to compare mean of two continuous normally distributed variables and the Mann–Whitney U test was run to determine mean of two continuous non-normally distributed variables

*Abbreviations: MSV* Microscopic varicocelectomy, *SP* Spontaneous pregnancy, *BMI* Body mass index, *TPMSC* Total progressively motile sperm count. TPMSC = total sperm number (10^6^/ejaculate) × progressive motility (PR, %); PR, progressive (a + b motility); *DFI* DNA fragmentation index

**Table 3:** Multivariate binary logistic regression of status of spontaneous pregnancy after MSV

Intercept and Variable	β	95% CI	OR	95% CI	*P*
Intercept	-22.659	-31.347, -13.971	1.44 × 10^–10^	2.43 × 10^–14^, 8.56 × 10^–7^	< 0.001
Age, female partners (years)	-0.284	-0.461, -0.107	0.753	0.631, 0.899	0.002
Diameter of veins (left, mm)	0.979	0.065, 1.894	2.663	1.067, 6.645	0.036
Baseline TPMSC (10^6^)	1.557	1.042, 2.071	4.743	2.836, 7.931	< 0.001
Increased TPMSC (10^6^)	0.556	0.331, 0.781	1.743	1.392, 2.183	< 0.001
**Area under ROC curve**					
Development Dataset	0.975	0.956, 0.994			< 0.001
Validation Dataset	0.987	0.970, 1.000			< 0.001

The β coefficient, odds ratio, and 95% confidence interval were measured through binary logistic regression

*Abbreviations: MSV* Microscopic varicocelectomy, *OR* Odds ratio, *CI* Confidence interval, *TPMSC* Total progressively motile sperm count. TPMSC = total sperm number (10^6^/ejaculate) × progressive motility (PR, %); PR, progressive (a + b motility); *ROC* Receiver operating characteristic curve

**Fig. 3:**
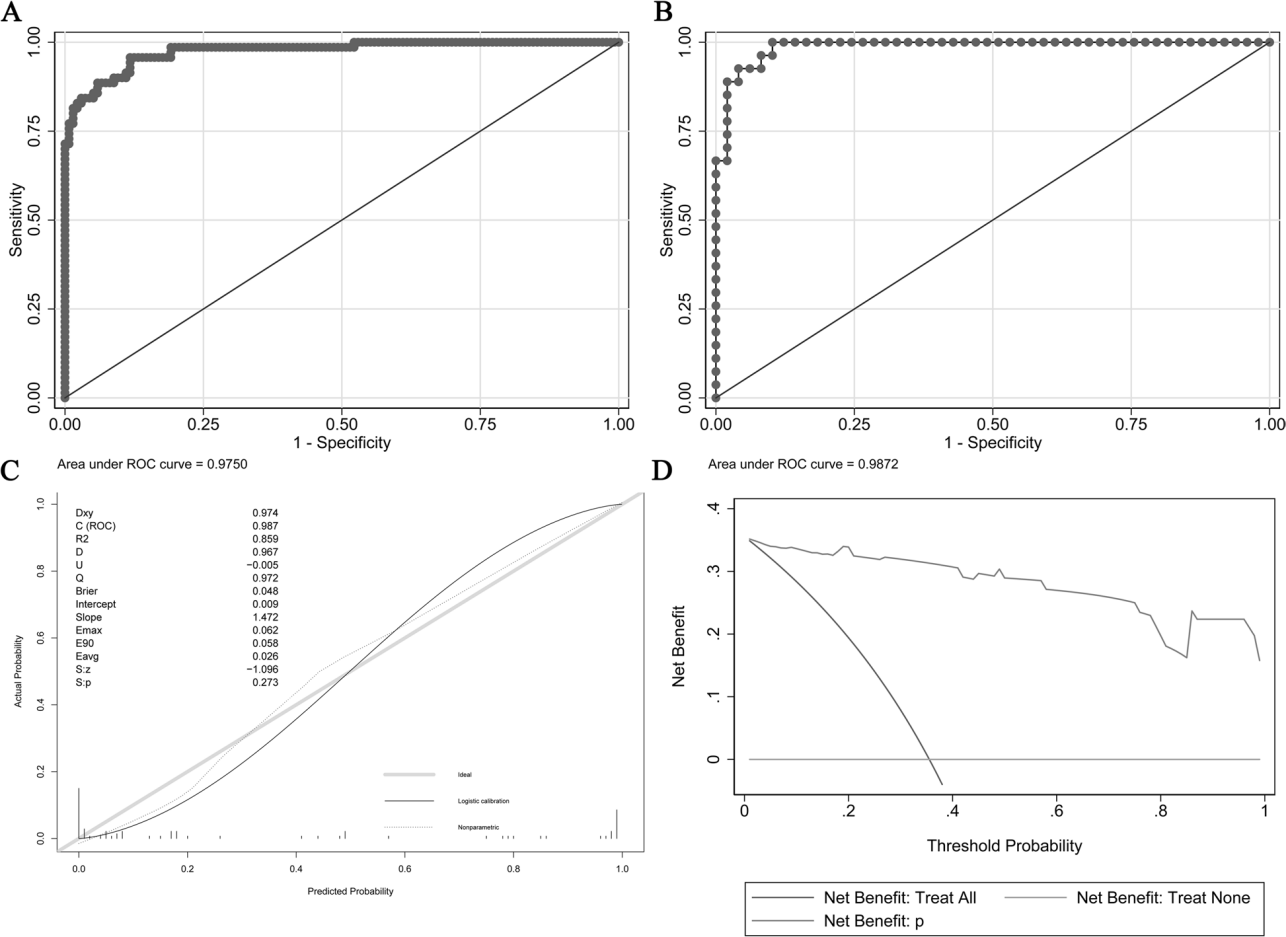
Discrimination, calibration, and decision curve analysis for the model. **A** ROC in the development cohort. **B** ROC in the validation cohort. **C** Calibration plot. **D** Decision Curve Analysis

The development and validation cohorts' AUROC values were 0.9750 and 0.9872, respectively, and the probability cutoff value in this model was 18.8%, with a sensitivity of 95.71% and specificity of 88.24% (Table 3 and Fig. 3). The calibration unreliability test statistic was -0.005 with a *p* value of 0.273; Emax and Eavg values were 0.062 and 0.026, respectively. The H–L chi-square statistic was 1.88, with a *p* value of 0.9972, indicating that the calibration was satisfactory. The decision curve revealed that when a patient's threshold probability ranged from 0 to 100%, using this nomogram to predict SP following MSV was more helpful than using either the treat-all-patients or treat-none schemes. The net benefit was comparable within this range (Fig. 3).

**Fig. 4:**
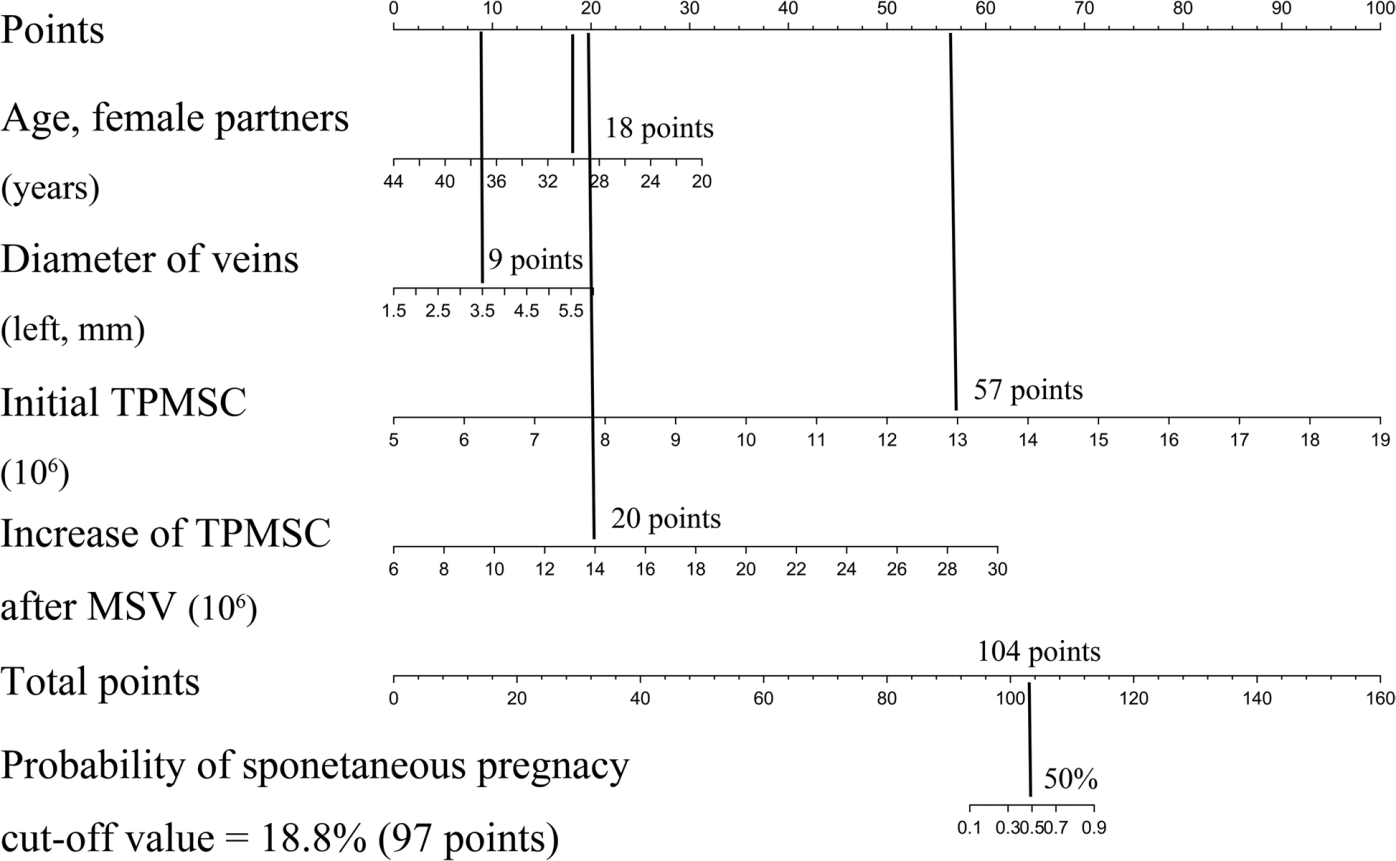
Example of nomogram to predict spontaneous pregnancy after MSV

By drawing a line straight upward to the Points axis, each clinicopathological feature matched to a specific point. After obtaining the sum of the points on the Total Points axis, a line was drawn straight down to the probability axis to determine the sum representing the probability of SP. For example, female partners are 30 years old (18 points), veins are 3.5 mm wide (9 points), baseline TPMSC is 13 × 10^6^/ml (57 points), and TPMSC increases after MSV are 14 × 10^6^/ml (20 points). This patient received a 104-point score, and the probability of SP after MSV was estimated to be at 50%. (the cutoff value was 18.8%, see details in Fig. 4). This estimated result could be useful in treatment plan decision-making.

During the follow-up period, only one patient suffered from hydrocele after surgery in the development cohort; no patient experienced testicular atrophy, scrotal hematoma, or wound infection.

## Discussion

The exact association between varicocele and reduced male fertility is unknown. Several hypotheses attempted to provide an explanation, such as hypoxia and hemostasis, increased scrotal temperature, autoimmunity, adrenal metabolite reflux, and increased oxidative stress [1]. However, it remains unclear why a minimum of one-third infertile men do not report improvement in semen parameters, and more than half of them do not experience fertility recovery post varicocelectomy [20, 21], and which patients can benefit from varicocelectomy. Therefore, this study aimed to develop and validate a nomogram for predicting SP post MSV in infertile men with abnormal semen parameters, based on a large cohort. Our findings suggest that younger female partners, larger diameter of veins, higher initial TPMSC, and increased TPMSC were significant predictors of SP in infertile men post MSV.

TPMSC was a combined indicator calculated from total sperm count × ratio progressively motile. This study found that baseline TPMSC positively correlated with SP post MSV. The baseline TPMSC was significantly higher in patients who reported SP than in those who did not recover from infertility (13.48 million vs. 9.32 million, *p* < 0.001). These findings were supported by Wang et al. [22], who conducted a meta-systemic study of nine studies, which showed that the SP rate was higher in patients who presented a higher baseline TMSC post varicocelectomy. SP rate was the highest (55.4%) in the TMSC > 20 million group, and lowest in the TMSC < 1.5 million group (16.0%). Another critical predictor for fertility recovery is the increase of TPMSC post MSV. Shomarufov et al., [14] who conducted a retrospective study on 93 infertile men with varicocele and pathozoospermia who underwent MSV, also identified that postoperative increase in TPMSC were predictors of SP post surgery. Furthermore, they found that 50% of the treated patients had an increase in TPMSC by 12.5 million. However 81% of patients who reported pregnancy achieved the same result. Therefore, the combined indicator of TPMSC should be used when assessing the predicted SP of planned varicocelectomies.

In this study, the diameter of veins (measured by ultrasound) played a significant role in predicting SP post varicocelectomy. These results were supported by Palmisano et al.’s findings [23], based on a retrospective study that recruited 228 patients who underwent unilateral MSV, that higher USVG (grade 3 above) patients were more likely to benefit from surgery. In our opinion, the US grading is superior to a palpable examination and more related to the treatment outcome because the physical examination is often ambiguous due to the subjective nature and is mainly dependent on the surgeon's experience. It is of limited value in obese patients or those with high-located testes and a history of surgery in the inguinal area. However, several studies found no association between the grade of varicocele and SP [15, 24]. Another significant predictor was the female partner's age which is the most important variable influencing outcomes in assisted reproduction [25]. Previous evidence indicated that compared to a 25-year-old woman, fertility reduces further with age: to 50% (35 years), 25% (38 years), and < 5% (40 years). García et al. [26] also found that the most significant decrease in fertility occurred in women > 35 years, thus making age one of the main risk factors for infertility. On the contrary, Peng et al. [15] and Zhang et al. [24] concluded that the female partner's age was unrelated to SP post surgery. This discrepancy could be explained by the inclusion criteria, surgical methods, and study design differences. It also indicates the difficulty of determining predictive factors for a successful outcome post varicocelectomy.

The study has several limitations. First, it uses a retrospective design. Second, some potential predictors are not included, such as anti-sperm antibodies; however, they are not widely accepted in clinical practice. Third, laparoscopic or open techniques were not included. This was because microscopic surgery is the gold standard in varicocelectomy. Fourth, the inability to obtain accurate data on the health condition of female partners because this information was completed by men in the survey. Fifth, there was a brief follow-up period (6–12 months). However, current evidence indicates that the average time to improve semen parameters is approximately two spermatogenic cycles [27], with SP occurring between six and 12 months post varicocelectomy [15]. This is the first nomogram for predicting the SP in infertile men post MSV by adjusting extensive confounding factors (including the age of female partners, life habits, surgical approach, and the number of ligation veins) based on a large cohort. This nomogram was successfully validated. Further largescale multi-center prospective studies are necessary to validate this predictive model and confirm its generalizability and clinical applicability.

## Conclusion

Our findings indicated that younger female partners, larger diameter of veins, higher initial and increased total progressively motile sperm count were significant predictors of spontaneous pregnancy in infertile men, post microscopic varicocelectomy. This nomogram may assist in individual decision-making on the treatment strategy of varicocele preoperatively and improve the treatment outcome.

## Data Availability

The datasets used and/or analysed during the current study available from the corresponding author on reasonable request. Lina Liu, Jia Li, Gang Liu, Chunyu Pan, Song Bai, Yunhong Zhan, Liping Shan. Liping Shan and Yunhong Zhan.

## Abbreviations

MSV: Microscopic varicocelectomy
SP: Spontaneous pregnancy
BMI: Body mass index
TPMSC: Total progressively motile sperm count
DFI: DNA fragmentation index
OR: Odds ratio
CI: Confidence interval
ROC: Receiver operating characteristic curve
